# Conductive PEDOT:PSS-Based Organic/Inorganic Flexible Thermoelectric Films and Power Generators

**DOI:** 10.3390/polym13020210

**Published:** 2021-01-08

**Authors:** Dabin Park, Minsu Kim, Jooheon Kim

**Affiliations:** School of Chemical Engineering & Materials Science, Chung-Ang University, Seoul 06974, Korea; dragoo@naver.com (D.P.); alstn275@gmail.com (M.K.)

**Keywords:** thermoelectric, silver selenide, nanowire, poly(3,4-ethylenedioxythiopene)-poly(4-styrenesulfonate)

## Abstract

We present a simple thermoelectric device that consists of a conductive poly(3,4-ethylenedioxythiophene):poly(styrenesulfonate) (PEDOT:PSS)-based inorganic/organic thermoelectric film with high thermoelectric performance. The PEDOT:PSS-coated Se NWs were first chemically synthesized in situ, and then mixed with an Ag precursor solution to produce the PEDOT:PSS-coated Ag_2_Se NWs. The PEDOT:PSS matrix was then treated with dimethyl sulfoxide (DMSO) prior to the production of flexible PEDOT:PSS-coated Ag_2_Se NW/PEDOT:PSS composite films with various weight fractions of Ag_2_Se via a simple drop-casting method. The thermoelectric properties (Seebeck coefficient, electrical conductivity, and power factor) of the composite films were then analyzed. The composite film with 50 wt.% NWs exhibited the highest power factor of 327.15 μW/m·K^2^ at room temperature. The excellent flexibility of this composite film was verified by bending tests, in which the thermoelectric properties were reduced by only ~5.9% after 1000 bending cycles. Finally, a simple thermoelectric device consisting of five strips of the proposed composite film was constructed and was shown to generate a voltage of 7.6 mV when the temperature difference was 20 K. Thus, the present study demonstrates that that the combination of a chalcogenide and a conductive composite film can produce a high-performance flexible thermoelectric composite film.

## 1. Introduction

Due to continuing global concerns about environmental pollution and the sustainability of energy generation, the identification and exploitation of new clean energy resources is vital. In this respect, thermoelectric (TE) devices have demonstrated significant potential for use in energy harvesting based on their conversion of thermal energy to electrical energy via the movement of charge carriers and internal phonons [1,2,3,4,5,6]. In the development of these devices, the TE performance of a prospective material is calculated using the dimensionless figure of merit (*ZT*), given by the formula in Equation (1):

*ZT* = *S*^2^ · *σ* · *T*/*κ* (1)

where *S*, *σ*, *T*, and *κ* are the Seebeck coefficient, electrical conductivity, absolute temperature, and thermal conductivity of the materials, respectively. A high figure of merit can be achieved by producing a high power factor (*PF* = *S*^2^ · *σ*) and a low thermal conductivity. The recent research on high-performance TE devices has focused primarily on inorganic materials such as oxides (e.g., NaCo_2_O_4_, SrTiO_3_, and CaMnO_3_) [7,8,9,10], in particular bulk materials (e.g., lead antimony silver telluride) [11,12], and Te-based semiconductors (e.g., Bi_2_Te_3_, Sb_2_Te_3_, and PbTe) [13,14,15,16,17,18,19]. However, conductive polymers such as polypyrrole (PPy) [20,21], polythiophene (PTh) [22,23], and polyaniline (PANI) [24,25] have been used as alternatives to the aforementioned inorganic TE materials due to their environmentally friendly nature, ease of processing, and low raw material costs. In addition, because polymers have high flexibility, high electrical conductivity, and low thermal conductivity, they have been explored for use in the fabrication of flexible TE devices. For instance, Wang et al. [26] fabricated PPy/graphene/PANI ternary nanocomposites and achieved an outstanding power factor of ~52.5 μW/m·K^2^, which is much larger than that of the pristine PPy and PANI. Other researchers have prepared single-walled carbon nanotube (SWCNT)-poly(aniline-co-pyrrole) copolymers with advantageous TE properties using traditional oxidative polymerization without mechanical agitation [27]. In particular, the conductive polymer poly(3,4-ethylenedioxythiopene):poly(4-styrenesulfonate) (PEDOT:PSS) has been extensively studied and has been shown to exhibit an outstanding electrical conductivity after doping or adjustment of its oxidation levels. For example, Luo et al. [28] studied the dimethyl sulfoxide (DMSO) post-treatment of a PEDOT:PSS thin film and reported a high electrical conductivity of ~930 S/cm. Similarly, Kim et al. achieved an improvement in electrical conductivity (~620 S/cm) and a high power factor (~33 μW/m·K^2^) by treating PEDOT:PSS with DMSO. However, conductive PEDOT:PSS has a lower power factor than that of inorganic TE materials due to its low Seebeck coefficient. This is because the improved technology mentioned above generally reduces the Seebeck coefficient by moving the Fermi level to the edge of the conductor [29]. For this reason, numerous studies have been conducted to improve the TE properties of polymer-based materials by introducing inorganic TE materials with a high Seebeck coefficient (e.g., Bi_2_Te_3_, SnSe, and Cu_2_SnSe_3_) [30,31,32]. For instance, Ge et al. [32] produced a Cu_2_SnSe_3_/PEDOT:PSS composite via spark plasma sintering to improve the TE properties. In addition, Zhang et al. [33] added Bi_2_Te_3_ to the PEDOT:PSS matrix to enhance the Seebeck coefficient and obtain a much larger power factor than that of the pure conductive polymers.

Silver selenide (Ag_2_Se) is a narrow bandgap N-type semiconductor material that has low thermal conductivity and high electrical conductivity at room temperature. Ag_2_Se has two phases, α-and β-Ag_2_Se, with β-Ag_2_Se acting as a cubic superionic conductor at ~408 K. Several studies have reported the outstanding TE properties of Ag_2_Se [34]. For example, Perez-Taborda et al. [35] used pulsed hybrid reactive magnetron sputtering to prepare an N-type Ag_2_Se film that exhibited a high figure of merit (i.e., 1.2) at room temperature. However, although Ag_2_Se has been proven to have advantageous TE properties at room temperature, pristine Ag_2_Se has not yet been widely used in the development of flexible TE devices.

Recently, our group developed a facile method for preparing an Ag_2_Se-based flexible film with a PEDOT:PSS matrix [36]. The composite film was shown to exhibit an enhanced power factor of 178.59 μW/m·K^2^ at room temperature. However, this value remained much lower than that of the bulk Ag_2_Se. This relatively low power factor is related to the density of the previously-reported composite film, which is only ~70% of the theoretical density due to the presence of voids between the polymer matrix and inorganic fillers.

In the present study, a polymer-coated NW type filler was first synthesized in order to improve the power factor of the composite film. The change in the morphology of the filler was shown to result in a compact film (~85% relative density) after drop casting. The PEDOT:PSS-coated Ag_2_Se NWs were fabricated by first coating Se nanoparticles with the PEDOT:PSS and then allowing these to grow to produce PEDOT:PSS coated Se NWs. The NWs were then reacted with the Ag precursor to form the PEDOT:PSS-coated Ag_2_Se NWs. The proposed inorganic/organic composite film was then generated by drop-casting and the PEDOT:PSS-coated Ag_2_Se NW/PEDOT:PSS composite film with 50 wt.% Ag_2_Se was shown to exhibit a maximum power factor of 327.15 μW/m·K^2^ at room temperature.

In addition, a simple thermoelectric generator was manufactured using the synthesized composite film and was shown to provide an output voltage of 7.6 mV when the temperature difference was 20 K. These results demonstrate the potential of the composite material for application in flexible electronic devices.

## 2. Materials and Methods

### 2.1. Materials

Selenium(IV) oxide (SeO_2_, 99%), β-cyclodextrin (C_42_H_70_O_35_, 98%), ethylene glycol (EG, C_2_H_6_O_2_, 99.5%), silver(I) nitrate (AgNO_3_, 99%), and L(+)-ascorbic acid (C_6_H_8_O_6_, 99%) were purchased from Daejung Chemical & Materials Co. (Seoul, Korea). The PEDOT:PSS (Clevious PH 1000) was purchased from Heraeus Clevios GmbH (Leverkusen, Germany). The dimethyl sulfoxide (DMSO; (CH_3_)_2_SO, 99%), and ethanol (C_2_H_5_OH, 98%) were purchased from Sigma Aldrich (St. Louis, USA). All materials were used without further purification.

### 2.2. Synthesis of PEDOT:PSS-Coated Ag_2_Se NWs

Solution I was produced by adding SeO_2_ (0.5 g), β-cyclodextrin (5 g), and PEDOT:PSS (2 mL) were to deionized (DI) water (100 mL) in a round-bottomed flask under stirring. Solution II was produced by adding ascorbic acid (2.5 g) to DI water (100 mL) in another beaker. After each solution was completely dissolved, solution II was poured into solution I and the reaction maintained for 4 h. The composite was then centrifuged and washed separately with DI water and ethanol. The synthesized product was stored in ethanol (100 mL). Subsequently, ammonium hydroxide (10 μL) was added to the solution, which was then left to stand for 48 h at room temperature, allowing time for a flocculant precipitate of PEDOT:PSS-coated Se NWs to form. The precipitate was collected using centrifugation and redispersed in ethylene glycol (200 mL).

An Ag precursor solution was produced with a certain amount of AgNO_3_ and ascorbic acid in DI water (5 mL). The molar ratio of the ascorbic acid and AgNO_3_ was 1:3. This Ag precursor solution was then poured into the as-prepared Se NW solution. After vigorous stirring for 4 h at room temperature to allow complete reaction, the product was centrifuged and washed with DI water and ethanol several times. Finally, the synthesized PEDOT:PSS-coated Ag_2_Se NWs were dried in a vacuum oven at 333 K for 24 h.

### 2.3. Fabrication of the PEDOT:PSS-Coated Ag_2_Se NW/PEDOT:PSS Composite Film

First, an appropriate amount of DMSO was mixed with the PEDOT:PSS solution and sonicated for 1 h to form a 5 vol.% DMSO/PEDOT:PSS mixture. After that, various amount of PEDOT:PSS-coated Ag_2_Se NWs (10 wt.%, 20 wt.%, 30 wt.%, 50 wt.% and 70 wt.%) were dispersed in the DMSO-treated PEDOT:PSS solution, and the mixtures were ultrasonicated for 3 h. The final solutions were drop-cast onto a glass substrate to produce the composite film and then dried in a vacuum oven at 333 K for 24 h.

### 2.4. Device Preparation

The synthesized composite film was cut into strips (18 mm × 6 mm). The 8 strips were then pasted onto a polyimide film at intervals of ~5 mm. The strips were then connected with copper wire, and an Ag adhesive was applied to connect the legs in series. An automatic temperature control system was used to vary the temperature at the ends of the device. The temperature at the two ends was measured using thermocouples. A multimeter (SENIT, A830L) was used to measure the output current and voltage.

### 2.5. Characterization

Field-emission transmission microscopy (FE-TEM, JEM-2100F, ZEISS, Oberkochen, Germany) and field-emission scanning electron microscopy (FE-SEM, SIGMA) were used to determine the microstructure and morphology of the composites. In addition, X-ray spectroscopy (EDS, NORAN system 7, Thermo Scientific, Seoul, Korea) was employed to determine the elemental mapping of the samples. The crystal structure of the material was characterized via X-ray diffraction (XRD, New D8-Advance/Bruker-AXS, Massachysetts, USA) at 40 kV and 40 mA with a scan rate of 1°/s and a 2θ range of 5° to 70° under Cu K*α* radiation (0.154056 nm). The binding energy of the materials was investigated using X-ray photoelectron spectroscopy (XPS, Thermo, U.K. alpha) with a monochromatic Al Kα X-ray source (1486.6 eV). Thermogravimetric analysis (TGA, TGA-2050, TA Instruments, New Castle, USA) was used to investigate the thermal degradation of the sample in an N_2_ atmosphere at a heating rate of 10 K/min. A homemade device consisting of a pair of voltmeters and thermocouples was used to measure the Seebeck coefficient (*S*), which was determined based on the linear relationship between the thermal electromotive force (Δ*V*) and the temperature difference (Δ*T*) of the composite (*S* = Δ*V*/Δ*T*). A four-point probe method was used to measure the electrical conductivity (*σ*) of the composite, and a digital micrometer was used to measure its thickness. The charge carrier mobility (*μ*) and concentration (*n*) were investigated using Van der Pauw technique-based Hall-effect measurements (HMS-3000, Ecopia, AZ, USA). The density of the prepared film was determined by measuring the mass and geometrical dimensions of the film. Five replicates of the composite samples were used for each test to verify the reproducibility of the experiments. 

## 3. Results and Discussion

**Figure 1:**
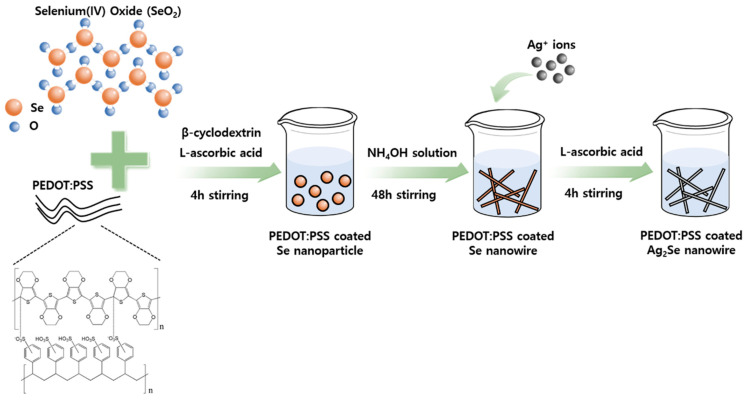
Schematic diagrams showing the preparation of the PEDOT:PSS-coated Ag_2_Se NWs.

The process for the synthesis of the proposed PEDOT:PSS-Ag_2_Se NW composite film is shown schematically in Figure 1. The fabrication of the Se NWs followed the same process as that described in previous studies [37]. To produce the PEDOT:PSS-Ag_2_Se NWs, first SeO_2_, PEDOT:PSS, and β-cyclodextrin were dispersed in (deionized) DI water. The resulting solution was then slowly poured into an L-ascorbic acid solution and mixed for four hours to synthesize PEDOT:PSS-coated Se nanoparticles (NPs). The reaction at this stage can be described by Equation (2):

SeO_2_ + 2C_6_H_8_O_6_ → Se + 2C_6_H_6_O_6_ + 2H_2_O (2)

To confirm the successful synthesis and surface morphology of the PEDOT:PSS-coated Se NWs, XRD, FE-SEM and FE-TEM were conducted and the results are presented in Figure S1. Thus, all of the XRD peaks for Se (Figure S1a) can be indexed as orthorhombic Se (JCPDS no. 86-2246), while the FE-SEM image (Figure S1b) confirms the presence of wire-like Se, and the high-magnification FE-TEM image (Figure S1c) clearly reveals the coating layer.

The resulting product was stored in ethanol with ammonium hydroxide to allow the growth of NWs with a preferential orientation of (100). This solution-based template-oriented fabrication approach is a simple strategy for 1D Se generation. At this stage, the PEDOT:PSS-coated Se NPs were transformed into NWs. The fabricated PEDOT:PSS-coated Se NWs and Ag precursor solution were then poured into ethylene glycol (EG) to produce Ag_2_Se NWs. The reaction process for the fabrication of Ag_2_Se NWs is as summarized by Equations (3) and (4):

2Ag^+^ + C_6_H_8_O_6_ → 2Ag + C_6_H_6_O_6_ + 2H^+^ (3)

2Ag + Se → Ag_2_Se (4)

Here, Ag^+^ is reduced by ascorbic acid and the newly formed Ag reacts with the Se to produce the Ag_2_Se NWs.

The successful synthesis of the PEDOT:PSS-coated Ag_2_Se NWs was demonstrated by XPS. The wide-range XPS spectrum is presented in Figure S2a, and the high-resolution core-level signals of S 2p, Ag 3d and Se 3d are presented in Figure S2b–d, respectively. Here, the Ag 3d_5/2_ and Ag 3d_3/2_ binding energies are observed at ~367.9 and 374.1 eV, respectively, and the peaks for Se 3d_5/2_ and Se 3d_3/2_ are visible at ~53.6 and 54.3 eV, respectively. These results are in good agreement with the previously reported data for the binding energies of Ag and Se in nanostructured Ag_2_Se [38]. For the PEDOT:PSS, the binding energies of 166-168 eV in the S 2p peaks are attributed to the PSS unit, while the PEDOT units are indicated by the binding energies of 163–166 eV. The observed binding energies agree with the previously reported values for S 2p, thus confirming the presence of PEDOT:PSS.

**Figure 2:**
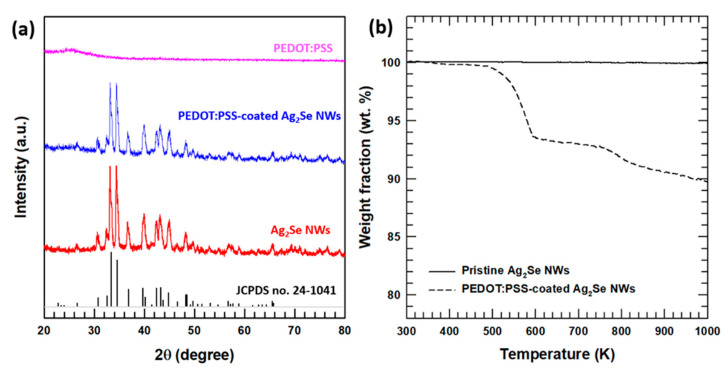
The characterization of the PEDOT:PSS-coated Ag2Se NWs and their synthetic intermediates: (**a**) the XRD patterns; (**b**) the TGA results.

The crystalline phases of the synthesized Ag_2_Se NWs, PEDOT:PSS-coated Ag_2_Se NWs, and pristine PEDOT:PSS were determined via XRD analysis, as shown in Figure 2a. Here, the XRD patterns of the Ag_2_Se NWs and PEDOT:PSS-coated Ag_2_Se NWs can be attributed to the orthorhombic crystalline phase of Ag_2_Se (JCPDS no. 24-1411), thus verifying the formation of well-defined Ag_2_Se (*a =* 0.705 nm, *b =* 0.782 nm, and *c =* 0.434 nm) [39].

Due to the relative weakness of the PEDOT:PSS diffraction peaks, these were not observed in XRD the pattern of the PEDOT:PSS-coated Ag_2_Se NWs. However, the presence of PEDOT:PSS in the composite material was confirmed via TGA, as shown in Figure 2b. Here, the pure Ag_2_Se NWs is seen to exhibit an outstanding thermal stability in an N_2_ atmosphere of up to ~1000 K, whereas the PEDOT:PSS suffers extensive thermal degradation at ~500 K, with a weight ratio of ~10 wt.%.

**Figure 3:**
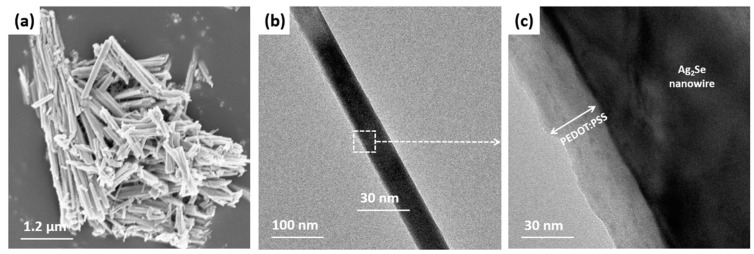
Imaging of the PEDOT:PSS-coated Ag_2_Se NWs: (**a**) FE-SEM; (**b**,**c**) low- and high-magnification FE-TEM.

The successful coating of PEDOT:PSS on the Ag_2_Se NWs was further demonstrated via FE-SEM and FE-TEM imaging, as shown in Figure 3. The wire-like structure of the PEDOT:PSS-coated Ag_2_Se NWs is clearly visible in the FE-SEM image in Figure 3a, while the coating is confirmed by the low- and high-magnification FE-TEM images in Figure 3b,c. In addition, the FE-TEM images of the pristine Ag_2_Se NWs provided in Figure S3 reveal their crystalline structure and the two indexed lattice planes at (200) and (002) with interplanar spacings of 0.35 nm and 0.25 nm, respectively [40]. Furthermore, uniform distributions of Ag, Se, and S atoms are observed in the FE-SEM images and the corresponding EDS maps of the PEDOT:PSS-coated Ag_2_Se NWs in Figure S4.

**Figure 4:**
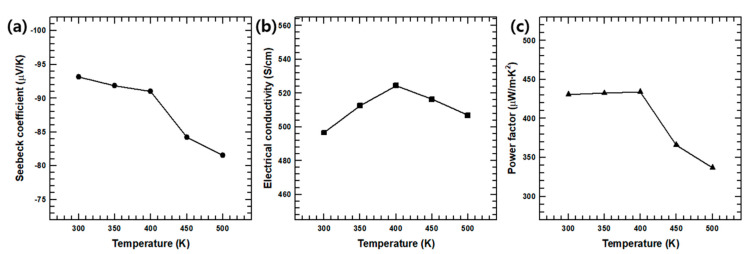
The temperature-dependent thermoelectric properties of the PEDOT:PSS-coated Ag_2_Se NWs: (**a**) Seebeck coefficient, (**b**) electrical conductivity, (**c**) power factor.

The temperature-dependent thermoelectric properties (Seebeck coefficient, electrical conductivity, and power factor) of the PEDOT:PSS-coated Ag_2_Se NWs are indicated in Figure 4. Here, the Seebeck coefficient is seen to decrease moderately with increasing temperature in Figure 4a, while in Figure 4b we see that the electrical conductivity initially increases with increasing temperature up to ~400 K, and subsequently decreases. The latter result is similar to the previously reported behavior of Ag_2_Se-based semiconducting materials [40]. These trends in the Seebeck coefficient and electrical conductivity reveal that the PEDOT:PSS-coated Ag_2_Se NWs exhibits a maximum power factor of 434.13 μW/m·K^2^ at 400 K.

The temperature-dependence of the Seebeck coefficient (*S*) and the electrical conductivity (*σ*) within the composite can be understood by analyzing the Hall measurement results in Figure S5 due to the relationships in Equations (5) and (6):

*σ* = *n* · *e* · *μ* (5)

(6) $$S=\;\frac{8\cdot \pi^{2}\cdot k_{B}{}^{2}}{3\cdot e\cdot h^{2}}\cdot m^{*}\cdot T\cdot {\left(\frac{\pi }{3\cdot n}\right)}^{\frac{2}{3}}$$

where *n*, *e*, *μ*, *k_B_*, *h*, and m* are the carrier concentration, electron charge, carrier mobility, Boltzmann constant, Planck constant, and effective mass of the carrier, respectively. The Ag_2_Se undergoes a phase transition from the semiconducting orthorhombic to the superionic cubic phase near 408 K [41]. The sudden changes in the TE properties and Hall measurement results between 390 and 420 K are due to this phase transition. Thus, the carrier mobility initially increases as the temperature is increased from 300 to 360 K, and subsequently decreases (first gradually and then more rapidly) as the phase transition temperature is approached.

**Figure 5:**
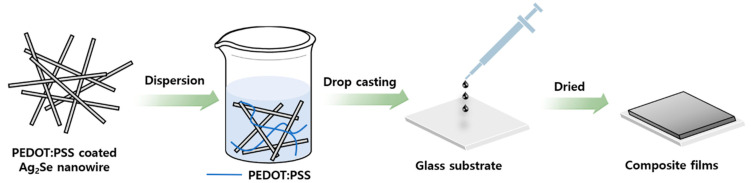
A schematic diagram of the preparation process for the PEDOT:PSS-coated Ag_2_Se NW/PEDOT:PSS composite films.

As shown schematically in Figure 5, the PEDOT:PSS-coated Ag_2_Se NWs were subsequently added to a solution of PEDOT:PSS in 5 vol.% DMSO, followed by drop-casting and drying to produce the composite film.

**Figure 6:**
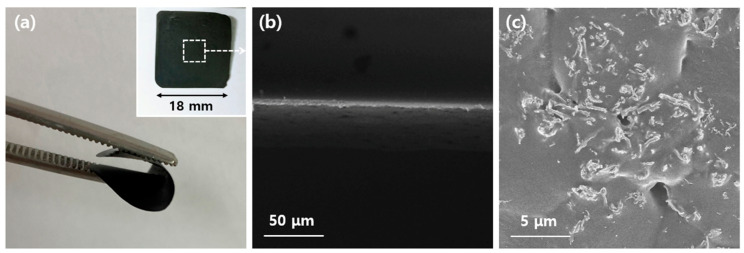
Imaging of the PEDOT:PSS-coated Ag_2_Se NW/PEDOT:PSS composite films: (**a**) digital photograph showing the bendable property; (**b**,**c**) FE-SEM images.

The characteristics of the resulting PEDOT:PSS-coated Ag_2_Se NW/PEDOT:PSS composite film are shown in Figure 6. Here, the film is seen to be black in color and square in shape, with a length of ~18 mm (inset, Figure 6a), and highly flexible (main image, Figure 6a), thus indicating its suitability for use in flexible TE modules. Further, the cross-sectional and surface FE-SEM image in Figure 6b,c reveal the uniform distribution of the PEDOT:PSS-coated Ag_2_Se NWs within the PEDOT:PSS matrix.

**Figure 7:**
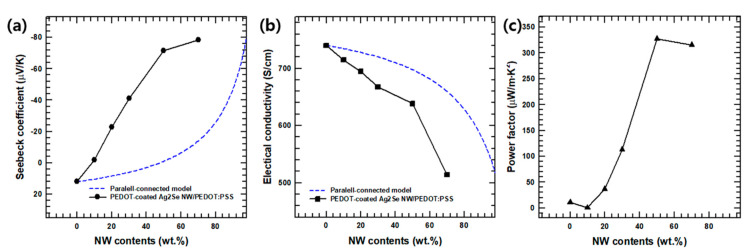
Analyses of the TE properties of the PEDOT:PSS-coated Ag_2_Se NW/PEDOT:PSS composite films with various NW contents according to the parallel connected model (**a**) Seebeck coefficient, (**b**) electrical conductivity (**c**) power factor values.

To investigate the potential of the composite film for use in TE devices, the TE properties of films containing various weight fractions of NWs (10 wt.%, 20 wt.%, 30 wt.%, 50 wt.% and 70 wt.%) were analyzed at room temperature. The Seebeck coefficient (*S*) and electrical conductivity (*σ*) of complex organic/inorganic systems can be predicted using a parallel-connected model, as summarized by Equations (7) and (8):

(7) $$S_{c,p}\;=\;\frac{x_{s}\sigma_{s}S_{s}+\left(1-x_{s}\right)\sigma_{p}S_{p}}{x_{s}\sigma_{s}+\left(1-x_{s}\right)\sigma_{p}}$$

(8) $$\sigma_{c,p}=\;x_{s}\sigma_{s}+\left(1-x_{s}\right)\sigma_{p}$$

where *S_c,p_*, *S_s_*, and *S_p_* are the Seebeck coefficients of the parallel-connected model, the PEDOT:PSS-coated Ag_2_Se NWs, and the PEDOT:PSS film, respectively, *σ_c,p_*, *σ_s_*, and *σ_p_* are the electrical conductivities of the parallel-connected model, the PEDOT:PSS-coated Ag_2_Se NWs, and the PEDOT:PSS film, respectively, and *x_s_* is the volume fraction of the PEDOT:PSS-coated Ag_2_Se NWs. The curves obtained from Equations (7) and (8) are plotted in Figure 7a,b, and the input values are listed in Table S1.

In close agreement with the previous research [42], the Seebeck coefficient of the pristine PEDOT:PSS (0 wt.% NWs) is seen to be ~12.85 μV/K in Figure 7a, with the positive value indicating P-type conduction. However, with a filler content of more than 10 wt.%, the Seebeck coefficient becomes negative, thus indicating N-type semiconducting behavior of the composite film. Further, as the contents of the PEDOT:PSS-coated Ag_2_Se NWs increased, the absolute value of the Seebeck coefficient tends to increase, reaching a maximum of −78.29 μV/K at 70 wt.%. Meanwhile, the room temperature electrical conductivity of the composite film is seen to decrease with increasing PEDOT:PSS-coated Ag_2_Se NW contents in Figure 7b. This is because the electrical conductivity of the DMSO that was added to the PEDOT:PSS is higher than that of the PEDOT:PSS-coated Ag_2_Se NWs. Finally, due to the changes in the Seebeck coefficient and electrical conductivity of the composite films, the room temperature power factor is the highest (~327.15 μW/m·K^2^) with a PEDOT:PSS-coated Ag_2_Se NWs content of 50 wt.%, see Figure 7c.

The variations in the TE properties of the PEDOT:PSS-coated Ag_2_Se NW/PEDOT:PSS composite films with 50 wt.% and 70 wt.% NWs according to the number of bending cycles are presented in Figure S6. Thus, although both films exhibit a reduction in durability with an increasing number of bending cycles, this is relatively minor. Hence, the composite film can be considered to have outstanding durability.

**Figure 8:**
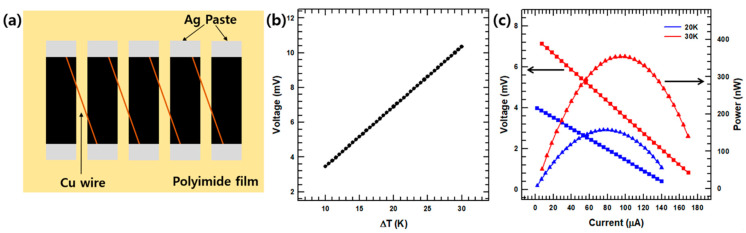
The structure and performance of the flexible TE prototype device: (**a**) schematic diagram of the TE device structure; (**b**) the open-circuit voltage at various temperature differences; (**c**) the output voltage and power versus current at temperature differences of 20 and 30 K.

To verify the high TE performance of the proposed composite film in practical application, a flexible TE prototype device was assembled. The device consisted of five strips of the composite film, as shown in Figure 8a. The open-circuit voltage and output power were measured using the homemade device shown in Figure S7. At a temperature difference (∆*T*) of 20 K, the prototype’s open-circuit voltage reached 7.6 mV (Figure S7b), which is similar to the theoretical value of 71.59 mV calculated according to Equation (9): [43]

*V_oc_* = *N* · │*S*│ · ∆*T* (9)

where *N* is the number of TE legs.

To measure the device’s output properties, a simple circuit was built using thermocouples and a voltage measurement device. A plot of the open circuit voltage against temperature difference is presented in Figure 8b. In addition, plots of the output voltage and power versus current at temperature differences of 20 K and 30 K are presented in Figure 8c. Here, the output voltage and output current are seen to be inversely related. The output power *P* was calculated using Equation (10): [43]

(10) $$P=I^{2}R_{load}={\left(\frac{V_{0c}}{R_{in}+R_{load}}\right)}^{2}R_{load}$$

where *I*, *R_load_*, and *R_in_* are the output current, load resistance, and internal resistance of the TE device, respectively. The maximum output power is obtained when *R_in_* equals *R_load_.* At a ∆*T* of 20 K (44.5 °C and 20.6 °C), the maximum output power *P_max_* was approximately 157.29 NW, corresponding to a load resistance of 91.8 Ω. In addition, for a ∆*T* of 30 K, *P_max_* reached 353.92 NW, and *R_load_* was 91.6 Ω.

Finally, the voltage was measured by applying different temperatures to both sides of the device. As shown in Figure S7, when the temperature difference was 20 K, the output voltage was 7.6.

## 4. Conclusions

In this report, PEDOT:PSS-coated Ag_2_Se NW/PEDOT:PSS inorganic/organic composite films with various NW contents were synthesized and their TE properties analyzed. The PEDOT:PSS-coated Ag_2_Se NWs were prepared with an Se template using in situ solution-phase synthesis. The synthesized NWs were then added to a PEDOT:PSS solution and drop-cast to form the flexible inorganic/organic composite film. The morphology of the composite film was analyzed using FE-SEM, FE-TEM, EDS, XPS, and XRD. The dramatically enhanced TE power factor of the proposed composite film is primarily due to the higher Seebeck coefficient and lower electrical conductivity. The addition of PEDOT:PSS-coated Ag_2_Se NWs to DMSO-treated PEDOT:PSS film also led to a higher power factor. However, when the NWs content was higher than 70 wt.%, a viable composite film could not be fabricated. Nevertheless, the composite films with 50 wt.% and 70 wt.% NWs exhibited outstanding durability after 1000 bending cycles. Finally, a simple flexible thermoelectric device was fabricated with five composite legs and was shown to generate a voltage of 7.6 mV at a temperature difference of 20 K. In summary, an efficient strategy was presented for designing and synthesizing high-performance inorganic/organic TE composite films by combining the high power factor of an inorganic filler with the high flexibility of a polymer.

## Data Availability

The data presented in this study are available on request from the corresponding author.

## Supplementary Materials

The following are available online at https://www.mdpi.com/2073-4360/13/2/210/s1, Figure S1: Characterization of the PEDOT:PSS-coated Se: (a) XRD pattern; (b) FE-SEM image; (c) FE-TEM image, Figure S2: XPS characterization of the PEDOT:PSS-coated Ag_2_Se NWs: (a) survey spectrum, and (b–d) high-resolution S 3p, (b) Ag 3d (c) Se 3d (d) spectra, Figure S3: FE-TEM images of the pristine Ag_2_Se NWs: (a) low magnification; (b) high magnification, Figure S4: Elemental characterization of the PEDOT:PSS-coated Ag2Se NWs: (a) FE-SEM image; (b–d) the corresponding EDS mappings of Ag (b), Se (c), and s (d) atoms, Figure S5: (a) Temperature dependent carrier concentration and (b) carrier mobility of PEDOT:PSS-coated Ag_2_Se NWs, Figure S6: The TE properties of PEDOT:PSS-coated Ag_2_Se NWs/PEDOT:PSS composites films with 50 and 70 wt.% of NWs as a function of bending cycles: (a) the Seebeck coefficient; (b) the electrical conductivity; (c) the power factor, Figure S7. The fabricated device: (a) schematic diagram of the electrical circuit; (b) measuring voltage difference due to the temperature difference between the two ends of the device; (c) an infrared thermal image showing a temperature difference of ~ 25 K, Table S1: Measured electrical conductivity, and Seebeck coefficient for the calculation of parallel-connected models.
