# Supplementary material for: Conductive PEDOT:PSS-Based Organic/Inorganic Flexible Thermoelectric Films and Power Generators
Source: Polymers (Basel). 2021 Jan 8;13(2):210. doi: 10.3390/polym13020210 (PMC7826913; doi:10.3390/polym13020210)
Supplement: Supplementary file 1 [file polymers-13-00210-s001.pdf]

Supplementary Information:

Conductive PEDOT:PSS-based organic/inorganic flexible thermoelectric films and power generators

Dabin Park, Minsu Kim and Jooheon Kim*

School of Chemical Engineering & Materials Science,

Chung-Ang University, Seoul 06974, Republic of Korea

*Corresponding author: jooheonkim@cau.ac.kr (J. Kim)

Supporting Information Contents:

1. Figures

2. Tables

1. Figures

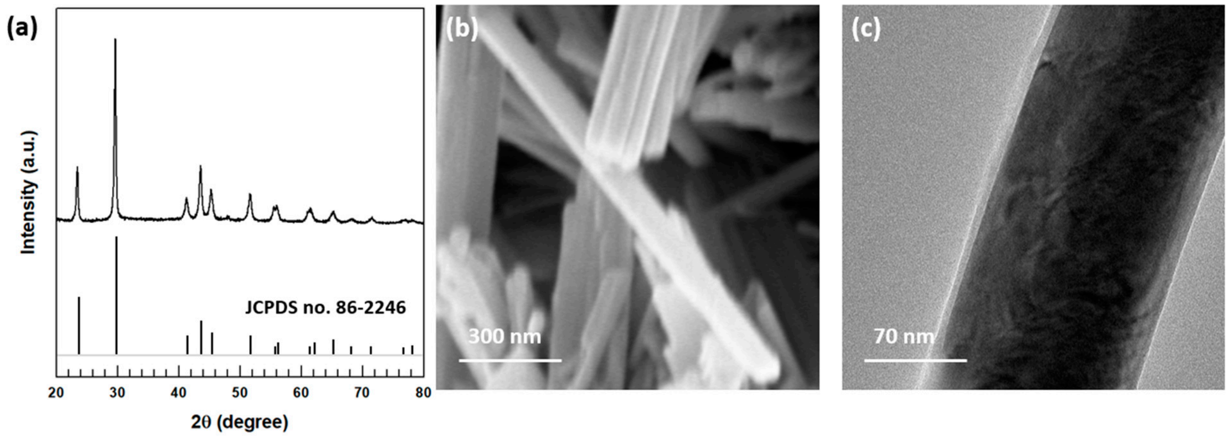

Fig. S1. Characterization of the PEDOT:PSS-coated Se: (a) XRD pattern; (b) FE-SEM image; (c) FE-TEM image.

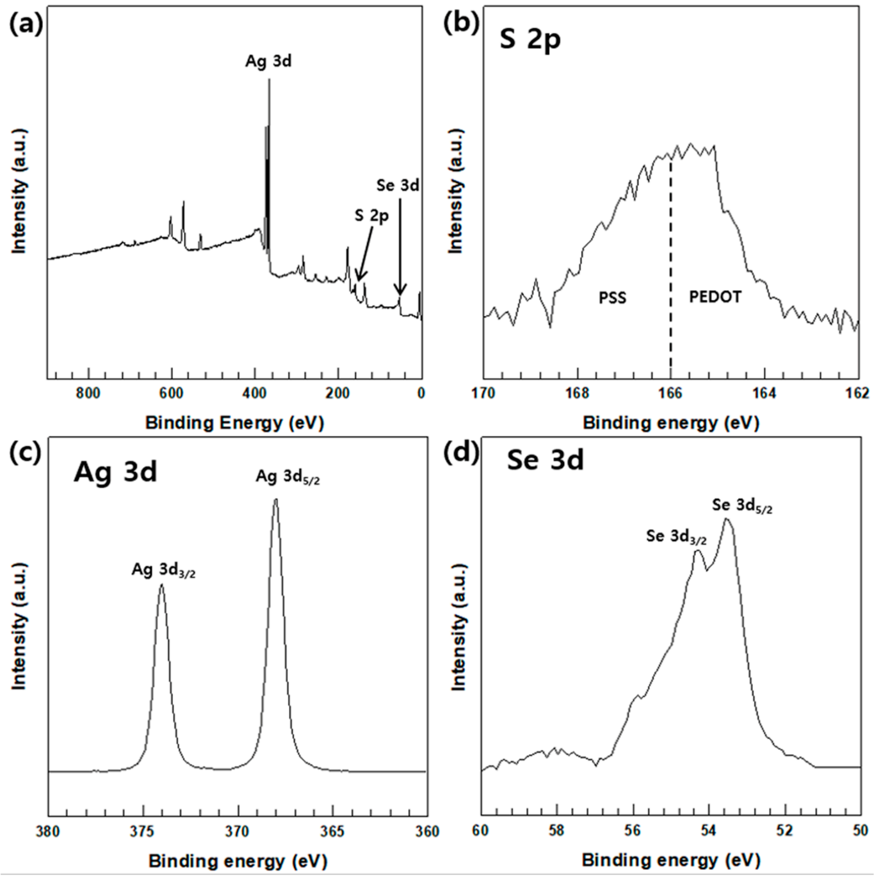

Fig S2. XPS characterization of the PEDOT:PSS-coated Ag_2Se NWs: (a) survey spectrum, and (b-d) high-resolution S 3p, (b) Ag 3d and (c) Se 3d (d) spectra.

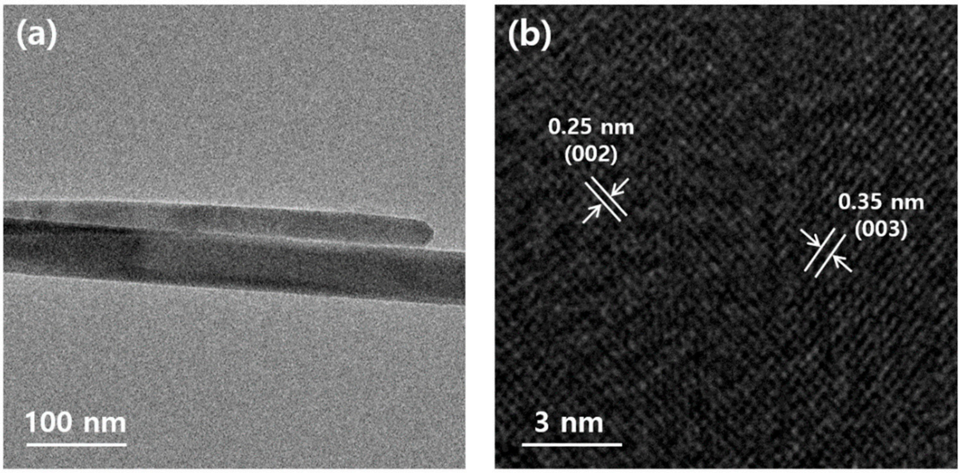

Fig. S3. FE-TEM images of the pristine Ag_2Se NWs: (a) low magnification; (b) high magnification.

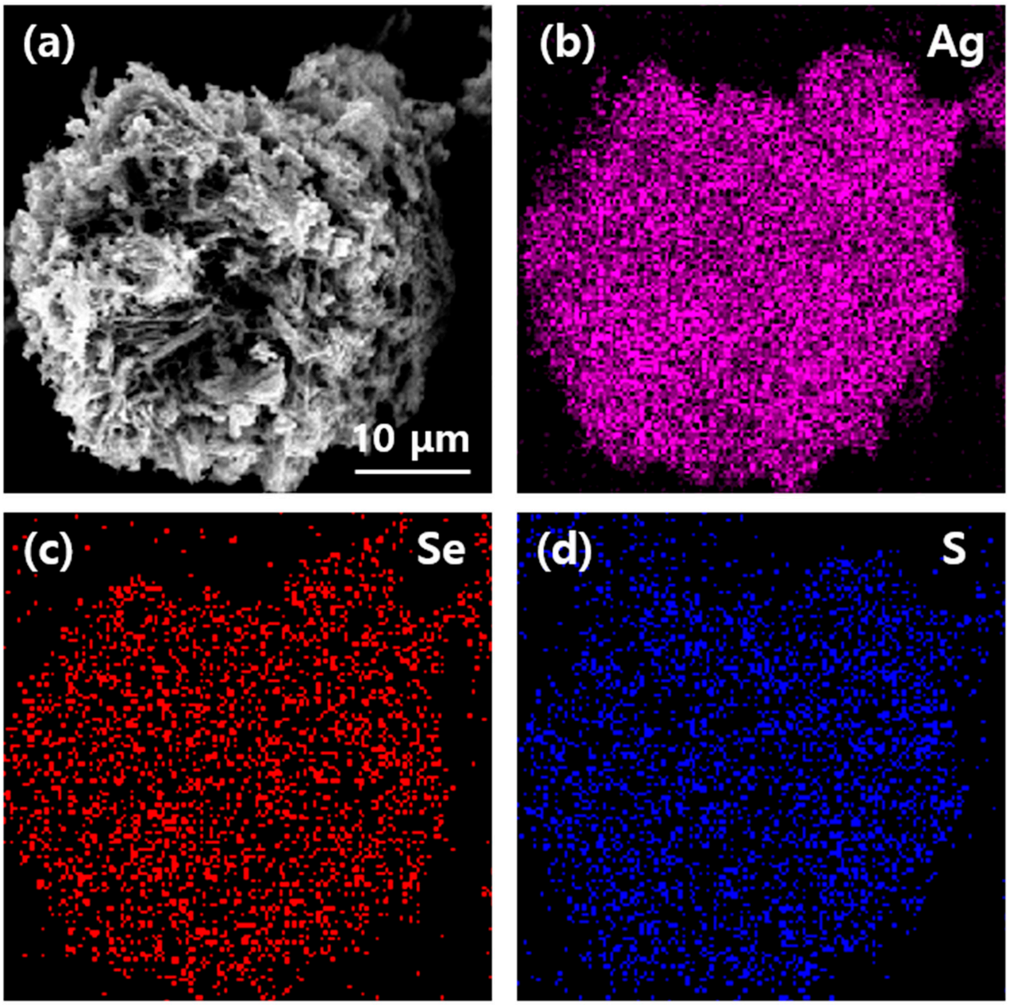

Fig. S4. Elemental characterization of the PEDOT:PSS-coated Ag_2Se NWs: (a) FE-SEM image; (b-d) the corresponding EDS mappings of Ag (b), Se (c), and S (d) atoms.

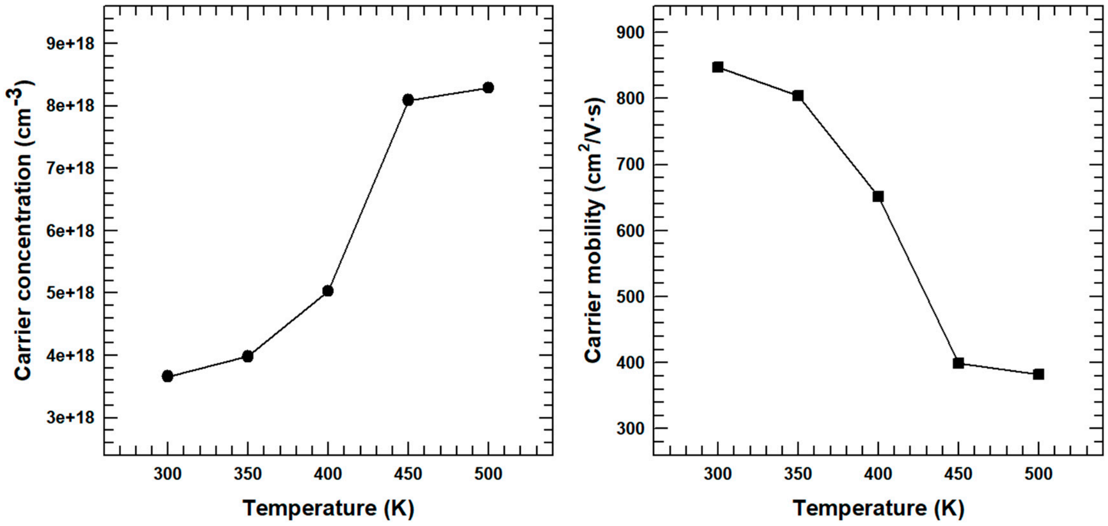

Fig. S5. (a) Temperature dependent carrier concentration and (b) carrier mobility of PEDOT:PSS-coated Ag_2Se NWs

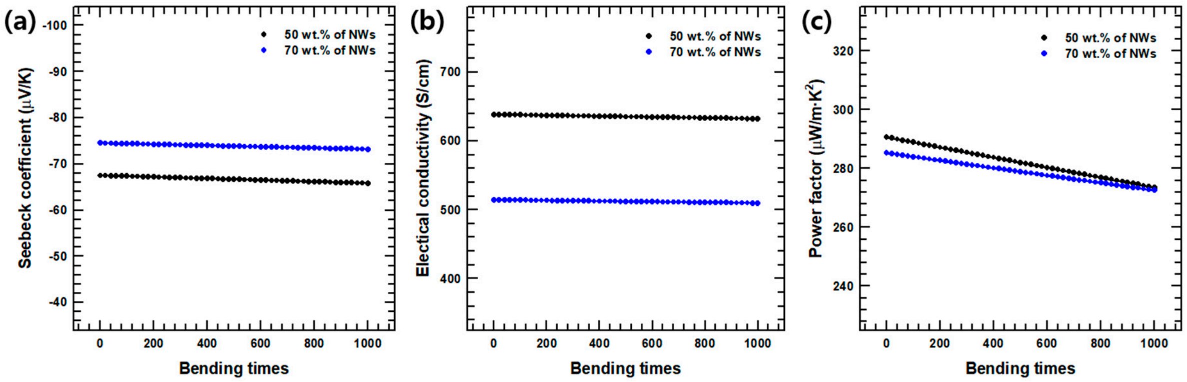

Fig. S6. The TE properties of PEDOT:PSS-coated Ag_2Se NWs/PEDOT:PSS composite films with 50 and 70 wt.% of NWs as a function of bending cycles: (a) the Seebeck coefficient; (b) the electrical conductivity; (c) the power factor.

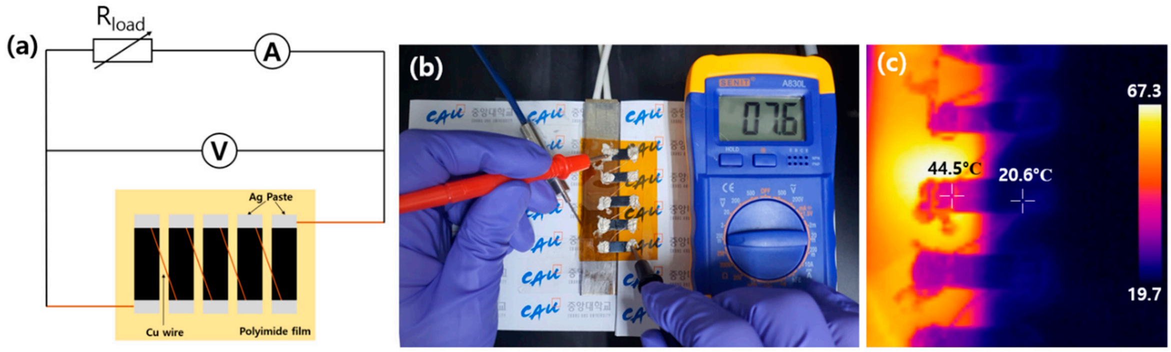

Fig. S7. The fabricated device: (a) schematic diagram of the electrical circuit; (b) measuring the voltage difference due to the temperature difference between the two ends of the device; temperature. (c) an infrared thermal image showing a temperature difference of ~20 K

2. Tables

	σ_s (S/cm)	σ_p (S/cm)	S_s (μV/K)	S_p (μV/K)
In this study	496.57	740	-93.14	12

Table S1. Measured electrical conductivity, and Seebeck coefficient for the calculation of parallel-connected models.
